# Comparing the Effects of Differential Learning, Self-Controlled Feedback, and External Focus of Attention Training on Biomechanical Risk Factors of Anterior Cruciate Ligament (ACL) in Athletes: A Randomized Controlled Trial

**DOI:** 10.3390/ijerph191610052

**Published:** 2022-08-15

**Authors:** Hadi Abbaszadeh Ghanati, Amir Letafatkar, Sadredin Shojaedin, Malihe Hadadnezhad, Wolfgang I. Schöllhorn

**Affiliations:** 1 Department of Biomechanics and Sport Injury, Faculty of Physical Education and Sports Sciences, Kharazmi University, Tehran 1571914911, Iran; 2 Department for Training and Movement Science, Johannes Gutenberg-University Mainz, 55122 Mainz, Germany

**Keywords:** anterior cruciate ligament injury, motor learning strategy, variability, biomechanics

## Abstract

The current study aimed to compare the possible effects of differential learning strategy, self-controlled feedback, and external focus of attention on kinetic and kinematic risk factors of anterior cruciate ligament (ACL) injury in athletes. Forty-eight male athletes from three sports of handball, volleyball and basketball were selected for this study and were randomly divided into four groups: differential learning (*n* = 12), self-control feedback (*n* = 12), external focus (*n* = 12), and control (*n* = 12) group. All groups followed the intervention for eight weeks with three sessions per week. Data were analyzed by means of 4 × 2 repeated measures ANOVA followed by post hoc comparison (Bonferroni) at the significance level of *p* ≤ 0.05. A significant group × time interaction and the main effect of time was found for most kinetic and kinematic variables. The main effect of the group was significant only at the knee abduction angle. Differential learning and external focus of attention methods positively reduced the kinetic and kinematic variables that are considered risk factors for ACL injury. However, the effect sizes (Cohen’s d) for the changes in most of the variables were larger for the differential learning group. Tailoring the boundary conditions that are based on the manipulations created in the exercise through variability and variety of movements associated with differential learning methods rather than repeating movements could reduce the risk of ACL injury.

## 1. Introduction

Athletes commonly encounter anterior cruciate ligament (ACL) injuries [1]. Approximately 70% of all ACL injuries are noncontact and occur during single-leg landings [2,3]. Although female athletes are relatively more likely to sustain ACL injuries, most absolute ACL injuries are observed in male athletes because they participate more frequently in athletic activities [4]. As a result of this injury, athletes experience restrictions in daily activities and reduced participation in sports [5]. Furthermore, ACL injuries are often accompanied by meniscal tears, (osteo-)chondral lesions, and an increased risk of premature knee osteoarthritis [6]. Events where athletes experience noncontact ACL injuries often show common biomechanical features as, e.g., exaggerated ground reaction forces (GRF) [7,8] during single-leg landing. In particular, the initial landing phase after a jump deserves special interest, as this is when most (31%) injuries occur [9]. Typically, it is assumed that during the landing, hip, knee, and ankle functional kinematics and dynamics are supportive for absorbing and distributing the forces adequately. A landing position with high GRFs and excessive “lower extremity valgus” (combined hip adduction and internal rotation, and knee abduction) seem to place athletes at higher risk of ACL injury [10,11,12]. Similarly, increased valgus during the landing process is considered a risk factor for ACL injuries. Athletes with dynamic knee valgus greater than 10 degrees were considered vulnerable to ACL injury [13]. Current rehabilitation programs do unsatisfyingly target proper movement control to prevent adverse movement patterns [14] and thereby minimize ACL injuries and their consequences. Although neuromuscular training programs have been applied to reduce the number of ACL injuries in some instances, there is still a need to improve their effectiveness to have a more marked impact on ACL injury rates [15,16]. Therefore, using motor learning strategies and a neuromuscular training program to perform exercises for adequate technique is investigated.

There are several approaches to motor learning. One of the most well-known approaches is the cognitive approach, where a learner mainly reaches the stage of automation by receiving augmented feedback and repeating the model technique as often as possible [17]. This approach uses instructions, role models, and augmented feedback combined with corrections and repetitive drills to instill ideal movement patterns. Thereby, variance in movement patterns is considered as noise that has to be avoided or at least to be minimized [18,19]. Self-controlled feedback (SF) is one of these methods where athletes request augmented feedback based on their needs, and the exercises are performed in a self-selective manner. A variant within the cognitive approach considered in this research is the external focus of attention [20,21] whereby an athlete’s attention is directed to landmarks outside the human body (e.g., “imagine sitting down on a chair when landing”). This approach supported advantageous prevention of ACL by using instructions that foster external focus [22,23]. For example, studies have found that using external focus (EF) of attention instructions and feedback in landing situations can effectively reduce injury. It also provided evidence that motor skills can be learned with the support of an external focus of attention to help ACL injury prevention [22]. In this regard, Benjamin et al. and Abbaszadeh Ghanati et al. found indications of advantages of using external focus strategies in neuromuscular programs [24,25]. However, besides evidence for individual attention strategies [26], it is not yet clear how these strategies compare to less cognitive approaches for injury prevention [24,27], which have been suggested recently and are considered to be useful in various dimensions of motor performance and are claimed to be useful for injury prevention [28,29]. 

One approach that has been considered effective is derived from system dynamics and the behavior of artificial neuronal nets. This approach considers learning as a self-organizing process that is initiated by destabilizing the system through conveying diffuse energy in the form of amplifying the system’s fluctuations stochastically [30]. This teaching method is the differential learning (DL) method which considers increased variable exercises as a basis for learning instead of repeating the movement. Thereby the exercises add stochastic perturbations to the to-be-learned movements pattern to ensure that the movement is not repeated and not corrected during the acquisition process [30,31]. Several studies have suggested that variability is important for exploration [32] and enhancing learning [33,34]. Methods that include variability in exercises facilitate movement adaptation as the exercise’s variability induces increases in the number of degrees of freedom (DOF) incorporated in movement control [35,36]. With increasing DOF, adaptation to a dynamic environment occurs, which leads to improved performance and reduced risk of injury [37]. Methods with variable content in training increase joint flexion angle and reduce the risk of ligament injuries by decreasing contact forces [38]. In the DL method, variability is essential for learning and spontaneous adaptation, allowing the individual to find their own flexible movement pattern to adapt to ever-changing situations [39,40]. The most extreme form of DL prescribes various exercises to individuals to demonstrate the vast number of possibilities, in contrast to the model-oriented repetition approaches for achieving the task goal. This extreme form of DL is based on information theoretic considerations whereby exact repetitions are completely redundant and do not contain any new information for the system for learning [41]. Feedback is omitted for two reasons. First, to initiate a true self-organization process in which no explicit information about the movement to be learned is given [42], and second because feedback has been studied only in conjunction with repetition learning, in which the learner has difficulty perceiving differences because his intention is to repeat the same thing, and because no advantages have been shown for variable training in the context of feedback-free training [43,44]. Instead of augmented feedback, athletes receive feedback from their own sensory system through the information provided by the differences in successive movements due to internal or external changes [45,46]. A recent study on these teaching methods provided evidence for being effective in preventing ACL injury as well [7]. Given the above contents, it can be claimed that the differential learning method for ACL injury prevention is at least as good as the other approaches (EF of attention and self-controlled feedback).

The purpose of the current study was to compare the effects of differential learning, self-controlled feedback, and external focus of attention on kinetic and kinematic risk factors of ACL in athletes. We hypothesized that in athletes who trained according to both EF of attention or DL methods, ankle, knee, and hip flexion angles, abduction and external rotation moments would increase more, and vertical GRF and knee abduction moments and angle would decrease more, compared with athletes who trained according to the SF method and the control group (CG).

## 2. Materials and Methods

### 2.1. Study Participants

A total of 42 athletes were involved in the final analyses: 16 handball, 14 volleyball, and 12 basketball players. Six athletes missed the follow-up (control group, *n* = 1; self-controlled feedback group, *n* = 2; the external focus of attention group, *n* = 1; and differential learning group, *n* = 2). Examination of demographic characteristics showed no statistically significant differences between the four groups in the mean and standard deviation of age, height, weight, body mass index, and sports experience (Table 1). 

**Figure 1:**
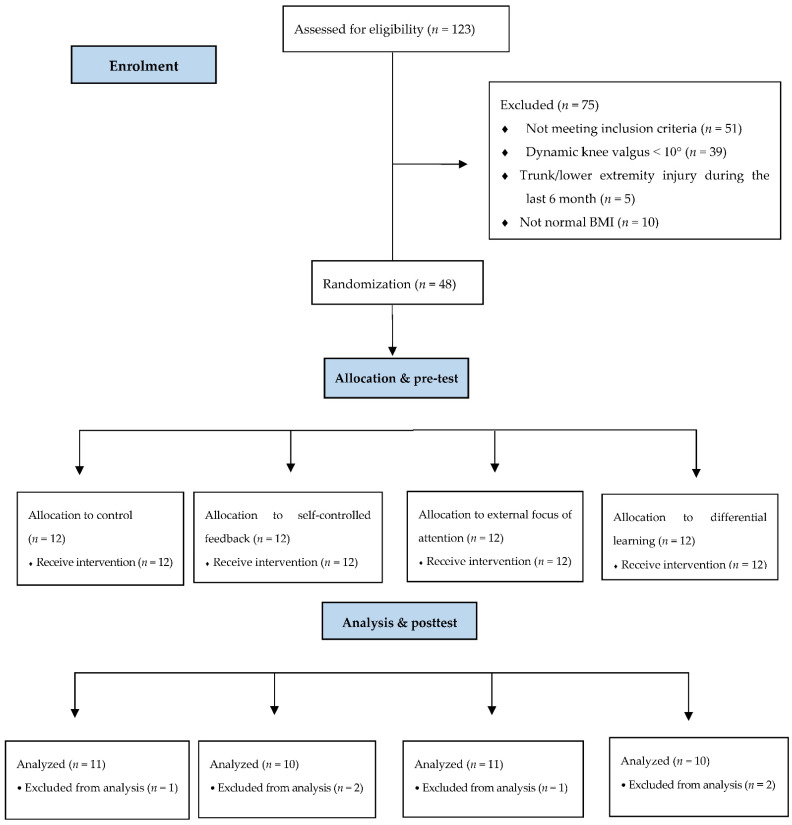
CONSORT flow diagram illustrating athletes’ enrollment, allocation, and analysis throughout study.

A randomized controlled trial design was used to complete the objective of this study. Upon enrollment, forty-eight competitive male athletes were randomly allocated by using the website http://randomizer.org (Social Psychology Network, Middletown, CT, USA, accessed on 22 June 2013), into one of four groups as follows: differential learning (DL) group (*n* = 12), external focus (EF) of attention group (*n* = 12), self-controlled feedback (SF) group (*n* = 12), and control group (CG) (*n* = 12) (Figure 1).

Before the study, the participants completed an informed consent form approved by the university. The Research Ethics Committees of the Iran institute of sports science approved the study (Approval IR.SSRI.REC.1400.1177). Inclusion criteria included athletes in three sports (handball, volleyball, and basketball), and (a) dynamic knee valgus more than 10 degrees during single-leg landing [13], (b) no injury to the trunk and lower extremity during the last six months, (c) age 20 to 25 years, and (d) BMI between 18.5–25 kg/m^2^. Exclusion criteria were: musculoskeletal abnormalities, history of fracture or dislocation of the hip, knee and ankle, history of lower back pain in the past year, and inability to perform functional tasks (visual and/or hearing, vestibular, and neurological impairments) [25,47].

Concealed allocation of the participants to the four groups was performed using a computer-generated block randomized table of numbers (1; DL, 2; EF, 3; SF 4; CG). The random numerical sequences were placed in sealed opaque envelopes in a box. Another researcher (blinded to the pretest assessment) opened the envelopes and proceeded with training according to the group allocation.

**Figure 2:**
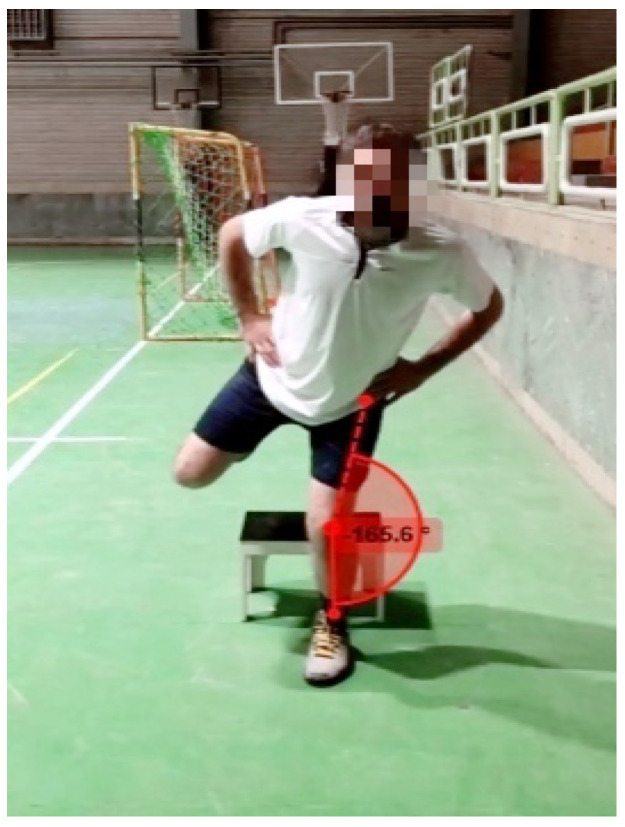
The single-leg landing test used to screen for dynamic knee valgus.

Dynamic knee valgus screening was measured by Kinovea software (Bordeaux, France, version 0.8.15) during a single-leg landing test. All subjects had to land on a single leg after dropping down from a height of 0.32 m with their dominant leg (Figure 2). The dominant leg was considered the leg the athlete preferred to land on following a jump [48].

**Figure 3:**
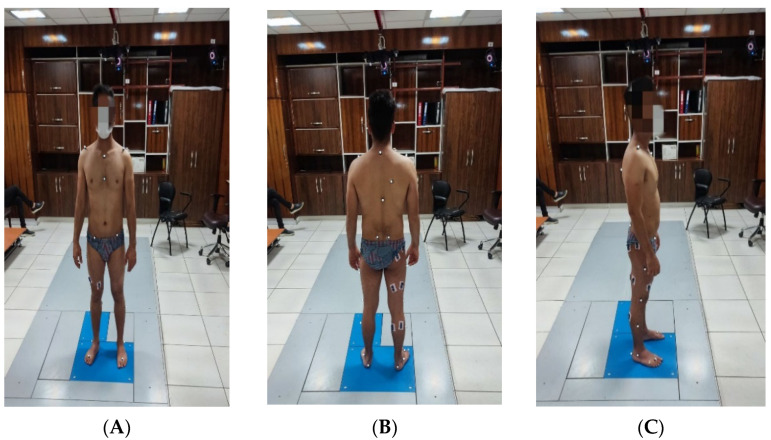
Depiction of the retroreflective marker placement in front (**A**), back (**B**), and side (**C**) views for kinematic data collection.

Retroreflective markers were attached to the trunk, pelvis, and lower extremities according to the plug-in gait marker set (Figure 3A–C) [49]. Next, a static calibration trial was conducted with athletes standing in the upright position. After completing the static calibration test, three-dimensional ground reaction forces (GRFs) at 1200 Hz (AMTI, Watertown, MA, USA), and an 8-camera motion capture system (Vicon Motion Systems, Oxford, UK) at 120 Hz were synchronously collected. Depending on the type of task and the markers installed on the lower and upper extremities, the volumes covered by the cameras on the ZX-, XY-, and ZY-planes were 3 m × 1.5 m × 1.5 m.

Force data were low-pass filtered using a fourth-order zero-lag Butterworth filter at 20 Hz and the marker data with 12 Hz. Initial contact and toe-off were determined when the vertical GRF first exceeded 10 N and first fell below 10 N before taking off. The period from initial contact to peak knee flexion angle was determined as the landing phase during the first landing. The peak values of hip abduction and external rotation moments, vertical and posterior GRFs, ankle dorsiflexion, knee and hip flexion, and knee abduction angles during the landing phase were calculated. Vicon Nexus software (Oxford, UK, version 33342.109.3.1) was used to determine the kinetic (force and moment) and kinematic of joints. For each athlete, the average of five successful trials was computed for each variable and used for statistical analysis. Data processing was performed using MATLAB engineering software (version 8.4, 2014b). Athletes with dynamic knee valgus greater than 10 degrees were considered vulnerable to ACL injury [13].

**Figure 4:**
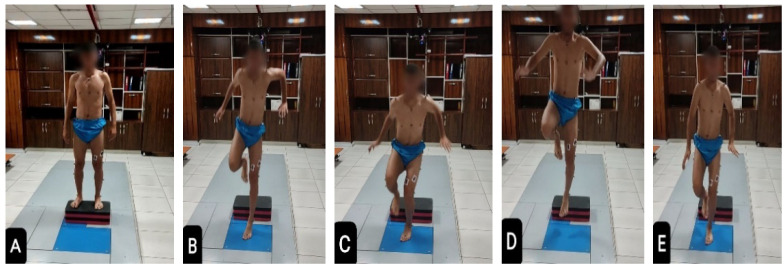
Single-Leg Vertical Drop Jump. Double-leg standing (**A**), single-leg drop landing (**B**), single-leg landing on force plate (**C**), immediate maximal vertical jump (**D**), landing again (**E**).

The single-leg vertical drop jump with the dominant leg started the same way as the previous test, but from a lower height, and instead of just landing, participants had to jump directly back up as high as they could after landing. The movement was divided into four stages as follows: (1) dropping from the 10-cm platform, (2) landing on the dominant leg, (3) performing an immediate maximal vertical jump and (4) landing again (Figure 4A–E) [48]. The trial was discarded and repeated if the athlete failed or obviously became out of balance, if the other leg touched the ground, or the athlete jumped off the box instead of dropping during the test. One minute of rest was given between each repetition to prevent fatigue.

For the intervention, all groups performed training programs for 8 weeks. During the first 6 weeks, athletes performed three training sessions per week, and during the last two weeks, they performed two training sessions per week. The athletes in the control group during the 8-week period completed their typical training regimen, like improving technique and sport-related skills. The training program of the DL, EF, and SF groups contained eight types of exercises: double-leg squats, walking lunges, single-leg squats, double-leg drop jumps, single-leg stance on an unstable surfaces, single-leg countermovement jumps, horizontal bounds, and single-leg standing long jumps (Table 2). Details of the type of exercises performed from weeks 1 to 8, as well as the repetitions and sets performed, are included in Table 2. The athletes in the EF, DL, and SF groups completed the same program presented in Table 2; however, they received different types of instructions according to the training characteristics of each group. In the case of the DL, although the central exercises were retained, these were provided with constant variations.

### 2.2. External Focus of Attention Exercise Program [23]

In double-leg squats, walking lunges, single-leg squad, and double-leg drop jump exercises, cones were positioned in line with the neutral knee position, and athletes pointed toes and knees toward the cones to promote proper alignment of the lower limb. For providing an unstable surface in single leg stance exercise, athletes stood on a small trampoline to challenge their balance; they were asked to hold a bar with straight arms, the hands at shoulder width to each other and in front of their body in a horizontal position in order to increase the forces related to anterior-posterior balance control. External targets (e.g., landmarks, cones, and hanging ball) were used to boost “neutral” alignment of the lower limb joints (e.g., ankle, knee and hip joints aligned vertically) or to use as a goal for the jump exercises (e.g., a small ball overhead that athletes tried to touch during jumping). During the exercises, verbal feedback instructions in the form of EF of attention learning strategies were given to the athlete (Table 3).

### 2.3. Differential Learning Exercise Program [5]

Athletes participated in a designed training program of exercises that were not repeated (not even in the sense of Bernstein’s “repetition without repetition”). Before each training session, the instructor designed a set of variants for each exercise (eight principle types of exercises provided in the training program). The two basic principles considered in the DL training program were no repetition and no correction. For example, in the double-leg jumping task, the nature of the jump was preserved, and executed under various external boundary conditions (e.g., on the sand, with and without shoes, dark situation, etc.) to provide a multitude of stimuli for the neuro-muscular system as a noisy basis for the training of the central and peripheral neuronal nets and in order to initiate a self-organizing process where no explicit information about the solution is provided. In this type of training, the exercises were arranged according to the instructor’s creativity, and the list of performed exercises was noted at the end of each training session (Table 4). 

### 2.4. Self-Controlled Feedback Exercise Program [5]

In the SF group, the athletes requested additional feedback based on their individual needs and the exercises were performed in a self-selected order. Eight types of exercises were arranged for eight weeks. By referring to the framework of cognitive approaches based on external feedback, an ideal pattern of skill was shown to the athletes. For example, if the trainer’s goal was to train jump-landing tasks, this skill was shown to athletes, and they were asked to copy and repeat it. If the athletes requested feedback, for example, the trainer instructed them on the proper jump-landing technique by verbal feedback, and if they did not ask for it, they would not get any feedback. In case of a feedback request from the athlete, the trainer could also encourage athletes with positive feedback to enhance feelings of success to optimize motor learning. It should be mentioned that each exercise of the eight available exercises had several variations; for example, athletes could choose three out of nine balance exercises in the order they preferred.

### 2.5. Sample Size Calculation and Statistical Analysis

Sample size calculation was performed using G*power software for windows (Düsseldorf, Germany, version 3.1.9.2), with an effect size of 0.25, an alpha level of 0.05 and a power of 0.80 was reached with 40 subjects [4]—40 subjects that means 10 subjects in each 4 groups. Considering the 20 percent loss from subjects missing follow-ups and possible missing data, a total of 48 subjects were calculated (12 subjects in each group).

Descriptive statistics consisted of means and standard deviations (SDs). An alpha level of <0.05 was used for statistical significance. The normal distribution of each kinetic and kinematics variable was assessed by the Kolmogorov–Smirnov test. All data were normally distributed. Levene test was used for homogeneity of the variance. The subject’s demographic characteristics values were compared using a one-way ANOVA statistical test. To determine differences between the four groups and time (pretest and posttest), 4 × 2 repeated measures ANOVA was directed, followed by post hoc comparison (Bonferroni) [4]. Within group factor (pretest to posttest) as a main effect of time and between-group as a main effect of the group were considered. Additionally, 95% confidence intervals (CI95%) were computed based on the adjusted group mean differences, and Cohen’s d effect size (ES) of 0.8, 0.5, and 0.2 were considered “large”, “moderate”, and “small” [50]. Data were analyzed in SPSS software (IBM Crop., Armonk, NY, USA).

## 3. Results

### 3.1. Kinematic Variables

Significant group × time interaction effects were found for the peak ankle dorsiflexion (F3,38 = 11.344; *p* = 0.001), peak knee flexion (F3,38 = 11.431; *p* = 0.001), peak hip flexion (F3,38 = 3.971; *p* = 0.015), and peak knee abduction angles (F3,38 = 25.510; *p* = 0.001) (*p* < 0.05). Additionally, significant main effects of time were found for the peak ankle dorsiflexion (F3,38 = 23.956; *p* = 0.001), peak knee flexion (F3,38 = 39.530; *p* = 0.001), peak hip flexion (F3,38 = 17.422; *p* = 0.001), and peak knee abduction (F3,38 = 84.703; *p* = 0.001) angles. The main effect of the group was significant only at the knee abduction angle (F3,38 = 3.441; *p* = 0.026). There were no significant between group differences in peak dorsiflexion and peak hip flexion angles. At peak knee flexion and abduction angles, significant differences were found between differential learning and control groups (*p* = 0.004, ES = 1.48; *p* = 0.001, ES = 1.99), and differential learning and self-controlled feedback groups (*p* = 0.013, ES = 1.29; *p* = 0.001, ES = 1.67). Additionally, significant differences in peak knee abduction angle were found between the external focus of attention and self-controlled feedback groups (*p* = 0.001, ES = 1.04). Post hoc test showed that differential learning and external focus of attention groups had significantly larger improvement in peak knee flexion angle (*p* = 0.001, ES = 1.19; *p* = 0.001, ES = 0.83), peak hip flexion angle (*p* = 0.001, ES = 0.99; *p* = 0.003, ES = 0.70), and peak knee abduction angle (*p* = 0.001, ES = 1.81; *p* = 0.001, ES = 1.19). Additionally, the differential learning group showed significant improvement in peak dorsiflexion angle (*p* = 0.001, ES = 1.01) (Table 5).

### 3.2. Kinetic Variables

Significant group × time interaction effects were found for the peak vertical GRF (F3,38 = 34.996; *p* = 0.001), peak hip abduction (F3,38 = 20.464; *p* = 0.001), and external rotation (F3,38 = 42.768; *p* = 0.001) moments and peak knee abduction (F3,38 = 4.962; *p* = 0.005) moment (*p* < 0.05). Additionally, significant main effects of time were found for the peak vertical GRF (F3,38 = 79.417; *p* = 0.001), peak hip abduction (F3,38 = 49.775; *p* = 0.001), and external rotation (F3,38 = 66.135; *p* = 0.001) moments and peak knee abduction (F3,38 = 26.179; *p* = 0.001) moment. There was no significant main effect on the group. Between-group differences in peak knee abduction moment and between, and within-group differences in posterior GRFs were not significant. Significant differences in peak vertical GRFs were found between differential learning and control groups (*p* = 0.010, ES = 1.60). Additionally, in peak hip external rotation moment significant differences were found between differential learning and control groups (*p* = 0.011, ES = 1.28), differential learning and self-controlled feedback groups (*p* = 0.005, ES = 1.57), and differential learning and external focus of attention groups (*p* = 0.016, ES = 1.35). Post hoc test showed that differential learning and external focus of attention groups have significant improvements in peak vertical GRFs (*p* = 0.001, ES = 1.86; *p* = 0.007, ES = 0.22), peak hip abduction moment (*p* = 0.001, ES = 1.12; *p* = 0.005, ES = 0.30), and peak hip external rotation moment (*p* = 0.001, ES = 1.16; *p* = 0.005, ES = 0.34). The self-controlled feedback group showed a significant decrease in peak vertical GRFs (*p* = 0.048, ES = 0.26). Additionally, differential learning (*p* = 0.001, ES = 1.14), external focus of attention (*p* = 0.021, ES = 1.05), and self-controlled feedback (*p* = 0.019, ES = 0.59) groups have significant improvement in peak knee abduction moment (Table 6).

## 4. Discussion

This study aimed to compare the effects of differential learning (DL) strategies, self-controlled feedback (SF), and external focus (EF) of attention on peak knee and hip flexion, ankle dorsiflexion and knee abduction angles, peak hip abduction and external rotation moments, peak knee abduction moment, and peak vertical and posterior GRFs of athletes. The main findings of the study showed statistically significant changes after DL and EF of attention interventions in reducing the potential kinetic and kinematic risk factors for ACL injury. The study hypothesis was confirmed: in the DL and EF of attention approaches, the joint flexion angles and hip moments increased more, and knee abduction moment and angle and vertical GRFs during landing decreased more than in the self-controlled feedback and control groups. Specially, the DL group appears to lead to even better performances than the EF group; this indicates the recommendation of using more rather than less variability during training. In the DL and EF of attention groups, knee abduction moment and angle and vertical GRFs were reduced more, and joint flexion angles and hip moments were increased more. Earlier findings are consistent with two parts of previous studies: First, the advantages of using variability in training [5,35,37,51] and reducing kinetic and kinematic risk factors of ACL injury [38]. Second, training with EF of attention instructions enhances neuro-muscular training in ACL injury prevention programs [25,52,53,54]. 

In general, over 70% of ACL injuries occur during single-legged ground contact without opponents’ influence, which requires the athlete to bear multiples of their body weight through one leg [55]. Evidence suggests increased coordination demands and higher forces in shorter times are needed when landing on a single-leg rather than double-leg [55,56]. Limitations in ankle dorsiflexion flexibility with the knee in extension are related to harmful landing mechanics [57]. Fang et al. showed that people with a small ankle dorsiflexion flexibility land with small knee angle during the jump-landing task [57]. During landing, hip, knee, and ankle movements help absorb GRFs. When these components are not synergistically effective in absorbing GRFs due to the small angles of the joints [58], tensional and rotational forces in the ACL ligament are increased by increasing GRF forces and are a major cause of ACL injury [59]. Further research requires further research on how the individual joint flexibilities must be coordinated. In terms of the DL method, increased fluctuations can help both increase joint angles and reduce GRFs [39]. When the joint flexion angles increase, adaptability and flexibility in the joint increase, and this reduces the extra GRFs applied to the ligaments by the muscle groups and prevents possible injuries [24]. Exercises with added noise in the kinematic position of a limb lead to desirable results compared to training in a fixed position with little variability [32]. If the execution of movements is repetitive because of the attachment of presumably single correct solutions, the same tissues will likely be under increased load each time. Adding optimum noise not only distributes the loads on the tissue at each execution much more and reduces the risk of fatigue and overuse but also allows the neuromuscular control more flexibility and, in consequence, decreases the risk of an injury [37].

Passive impairment in hip strength is just one of the several factors that clinicians use when assessing risk factors for ACL non-contact injury. Our findings suggest that the screening method for assessing the risk of ACL injury should include an assessment of active hip external rotational and abduction strength [60]. Furthermore, several in vitro studies have also shown that the knee abduction moment is a major contributor to ACL strain and considered to play an important role in the ACL injury mechanism [61,62]. Hence, in the present study, improving the hip and knee moments following the DL exercises can be considered a possible method for injury and overuse prevention [29].

According to the results, it can be concluded that motor learning methods have different effects on reducing the risk factors for ACL injury, and DL may be the most promising of the three applied methods (self-controlled feedback, external focus of attention feedback and differential learning). However, more studies with different designs are needed to provide further corroboration. In the original sense of Fisher’s statistics, the *p*-values smaller than 0.05 indicate that it is worth continuing research in this direction. DL strategy seems to help the individual to become more flexible and to be able to adapt to various boundary conditions in a shorter time more adequately [63]. When an athlete can cope with a greater number of DOF, the movement occurs more smooth, and the pressure from the ground reaction force is a kind of neutralized or distributed better by the muscles, tendons and ligaments; therefore, the person becomes less prone to injury [63,64]. Additionally, variability in training allows the athletes to explore and select more adequate solutions in accordance with the boundary conditions given by the external and internal situation, which leads to increased individual adaptation to the situation [35]. More variability during training sessions is considered functional. It increases individual movement coordination repertoire, enhancing dynamic environment adaptiveness, and probably reducing the risk of injury [37]. 

The current study has no claim for generalization. Nonetheless, in several areas, extended studies are encouraged to understand the mechanisms of ACL injuries further. First, our study only included male athletes [1] because we had better access to them, so the results of the study have to be tested for females, younger, elderly, untrained etc., and people with musculoskeletal impairments, higher BMI, or patients. In particular, other studies indicate that female athletes may respond differently to varying types of instruction [24]. Second, further studies are needed to clarify whether our findings can be extrapolated to other ACL injury-related kinematic and kinetic risk factors in other tasks such as cutting. Third, individual muscle activations should also be considered besides the kinematics and dynamics of the movements.

## 5. Conclusions

Both DL and EF of attention methods showed a positive effect on reducing possible kinetic and kinematic risk factors of ACL injury. However, the results of the present study consider the DL method and the role of variability in training to be very effective in these parameters. Additionally, tailoring training conditions based on the manipulations created in the exercise through variability and variety of movements, instead of repeating movements, may be effective in reducing the risk of ACL injury.

## Figures and Tables

**Table 1:** Demographic characteristics of the participants.

Characteristics	Control (*n* = 11)	Self-Controlled Feedback (*n* = 10)	External Focus of Attention (*n* = 11)	Differential Learning (*n* = 10)	*p* Value
Age (years)	23 ± 0.63	22.30 ± 1.49	22.36 ± 2.15	21.80 ± 1.61	0.390
Body mass (kg)	74.34 ± 7.03	76.83 ± 4.91	75.71 ± 7.46	73.79 ± 6.31	0.722
Body height (cm)	182.82 ± 7.56	185.70 ± 6.49	183.91 ± 8.11	184.80 ± 4.61	0.801
Body mass index (kg/m^2^)	22.20 ± 1.20	22.29 ± 0.37	22.39 ± 1.52	21.57 ± 0.88	0.342
Sports experience (years)	6.27 ± 3.10	7.40 ± 2.59	5.81 ± 2.31	6.70 ± 2.58	0.580

*p* value from one-way ANOVA test; significant difference (*p* < 0.05).

**Table 2:** Details of training program *^,†^.

Exercise	Week 1	Week 2	Week 3	Week 4	Week 5	Week 6	Week 7	Week 8
Double leg squats	3 × 6	3 × 6	—	—	—	—	—	—
Walking lunges	3 × 6	3 × 6	—	—	—	—	—	—
Single-leg squats	3 × 6	3 × 6	4 × 8	4 × 8	4 × 12	—	—	—
Double-leg drop jumps	—	—	3 × 6	4 × 10	4 × 12	—	—	—
Single leg stance on unstable surface	—	—	3 × 30 s	3 × 30 s	4 × 30 s	4 × 30 s	3 × 30 s	3 × 30 s
Single-leg countermovement jumps	—	—	3 × 6	3 × 8	4 × 8	4 × 10	3 × 8	3 × 6
Horizontal bounds	—	—	—	—	—	4 × 8	5 × 10	3 × 8
Single-leg standing long jumps	—	—	—	—	—	4 × 8	5 × 8	3 × 8

* Sets × repetitions or seconds for each exercise across the 8-week training program. ^†^ Athletes given 30–60 s of rest between sets.

**Table 3:** Educational instruction with external focus of attention.

Exercises	External Focus of Attention
Double leg squats	“While bending your knees, point your knees toward the cones and imagine you are going to sit on a chair while keeping a ball between your knees.” Note: Cones positioned in line with neutral * knee position.
Walking lunges	“While imagining you have a plank on your back, point your knee toward an imaginary point in front of you.”
Single-leg squats	“Stand on one leg and reach slowly toward the cone with your knee while bending your knee.” Note: Cone positioned in line with neutral * knee position.
Double-leg drop jumps	“Jump down, land on the markers on the floor, and point your toes and knees toward the cones.” Note: Cones positioned in line with neutral * knee positions drop from a 30-cm box
Single leg stance on the unstable surface	“Keep the bar horizontal.” Note: Athletes held a bar in front of them during exercise.
Single-leg countermovement jumps	“Jump as high as you can and touch the hanging ball.” Note: Ball included as an overhead goal; height adjusted per athlete
Horizontal bounds	“Push against the ground as forcefully as possible”
Single-leg standing long jumps	“Try to jump past the line.” Note: Target line provided; distance adjusted per athlete.

* “Neutral” knee position reflects vertical alignment of the hip, knee, and ankle joints.

**Table 4:** Examples of how to use differential learning to practice double-leg jumping.

Variation of the Double-Leg Jumping	Change of Environment Boundary Conditions
Jump as high as you can while jumping: “Before jumping, 3–4 bunny hops in different directions, skipping both legs then left and right legs, high knees, left and right high knees respectively, butt-kicks (both legs, left and right legs), zigzag movement, shuffle to the left and right.” “Make a jump with a full turn to the left and then to the right.” “While jumping: keep your arms behind your back, across the chest, raise the right arm, raise the left arm, circle (both arms, left and right arms), move the head to the right, to the left, close the right and left eyes in each jump respectively.” “While landing: one arm behind and the other one in front of you.” “Land with a very narrow or with a very wide stance” “Land on your toes”	“Exercises on the sand with and without shoes” “Exercises in a dark situation In a surrounding with the sound of music or with the noise from a spectator in a stadium or a virtual reality surrounding.”

**Table 5:** Kinematic variables of athletes during single-leg vertical drop jump.

Kinematic Variables	Group	Pretest Mean ± SD	Eight Weeks Mean ± SD	ES (CI_95%_) ^†^	*p* Value		
					Main Effect of Time	Main Effect of Group	Group × Time Interaction
Peak ankle dorsiflexion (°)	Control	17.68 ± 2.04	17.80 ± 2.17	0.05 (−0.77 to 0.89)	F = 23.956 *p* = 0.001 *	F = 0.691 *p* = 0.563	F = 11.341 *p* = 0.001 *
	Self-controlled feedback	17.58 ± 2.99	18.33 ± 2.93	0.25 (−0.62 to 1.13)			
	External focus of attention	17.84 ± 2.32	18.27 ± 2.49	0.17 (−0.65 to 1.01)			
	Differential learning	17.71 ± 2.24	20.44 ± 3.06 ^¥^	1.01 ^£^ (0.08 to 1.94)			
Peak knee flexion (°)	Control	49.83 ± 6.91	49.74 ± 6.27	0.01 (−0.84 to 0.82)	F = 39.530 *p* = 0.001 *	F = 2.737 *p* = 0.057	F = 11.431 *p* = 0.001 *
	Self-controlled feedback	49.15 ± 8.05	51 ± 7.67	0.23 (−0.64 to 1.11)			
	External focus of attention	52.29 ± 5.45	58.01 ± 8.05 ^¥^	0.83 ^£^ (−0.03 to 1.70)			
	Differential learning	51.66 ± 8.23	63.36 ± 11.11 ^¥,a,b^	1.19 ^£^ (0.24 to 2.14)			
Peak hip flexion (°)	Control	37.88 ± 8.92	37.95 ± 8.96	0.00 (−0.82 to 0.84)	F = 17.422 *p* = 0.001 *	F = 0.669 *p* = 0.576	F = 3.971 *p* = 0.015 *
	Self-controlled feedback	38.75 ± 8.33	40.74 ± 9.36	0.22 (−0.65 to 1.10)			
	External focus of attention	37.48 ± 9.75	44.85 ± 11.03 ^¥^	0.70 (−0.15 to 1.56)			
	Differential learning	38.11 ± 7.40	48.31 ± 12.40 ^¥^	0.99 ^£^ (0.06 to 1.92)			
Peak knee abduction (°)	Control	13.50 ± 3.06	13.73 ± 2.28	0.08 (−0.75 to 0.92)	F = 84.703 *p* = 0.001 *	F = 3.441 *p* = 0.026 *	F = 25.510 *p* = 0.001 *
	Self-controlled feedback	13.51 ± 2.75	12.34 ± 1.47	0.53 (−1.42 to 0.36)			
	External focus of attention	13.54 ± 3.07	10.22 ± 2.43 ^¥,d^	1.19 ^£^ (−2.10 to −0.29)			
	Differential learning	13.28 ± 2.44	6.86 ± 4.38 ^¥,a,b^	1.81 ^£^ (−2.85 to −0.77)			

Abbreviations: Results are presented as mean ± SD; *, statistically significant difference (*p* < 0.05); ^¥^, pretest to posttest significant difference; ^†^, effect size (95% confidence intervals); ^£^, large Cohen’s d effect size (0.8); results of Bonferroni post hoc test: ^a^ = significant difference between differential learning and control groups; ^b^ = significant difference between differential learning and self-controlled feedback groups; ^d^ = significant difference between external focus of attention and self-controlled feedback groups.

**Table 6:** Kinetic variables of athletes during single-leg vertical drop jump.

Kinetic Variables	Group	Pretest Mean ± SD	Eight Weeks Mean ± SD	ES (CI_95%_) ^†^	*p* Value		
					Main Effect of Time	Main Effect of Group	Group × Time Interaction
Peak vertical GRF (N/kg)	Control	32.35 ± 4.22	32.46 ± 4.26	0.02 (−0.80 to 0.86)	F = 79.417 *p* = 0.001 *	F = 1.069 *p* = 0.374	F = 34.996 *p* = 0.001 *
	Self-controlled feedback	31.09 ± 3.33	30.30 ± 2.66 ^¥^	0.26 (−1.14 to 0.61)			
	External focus of attention	31.54 ± 4.66	30.49 ± 4.82 ^¥^	0.22 (−1.05 to 0.61)			
	Differential learning	31.99 ± 3.05	26.98 ± 2.25 ^¥,a^	1.86 ^£^ (−2.92 to −0.81)			
Peak posterior GRF (N/kg)	Control	3.96 ± 0.85	3.97 ± 0.82	0.01 (−0.82 to 0.84)	F = 1.369 *p* = 0.249	F = 0.499 *p* = 0.686	F = 1.028 *p* = 0.391
	Self-controlled feedback	3.69 ± 0.74	3.69 ± 0.76	0.00 (−0.87 to 0.87)			
	External focus of attention	3.91 ± 0.90	3.90 ± 0.92	0.01 (−0.84 to 0.82)			
	Differential learning	4.02 ± 0.82	3.97 ± 0.82	0.06 (−0.89 to 0.77)			
Peak hip abduction moment (Nm/kg)	Control	2.91 ± 0.51	2.89 ± 0.51	0.03 (−0.87 to 0.79)	F = 49.775 *p* = 0.001 *	F = 1.226 *p* = 0.314	F = 20.464 *p* = 0.001 *
	Self-controlled feedback	2.77 ± 0.43	2.84 ± 0.45	0.15 (−0.71 to 1.03)			
	External focus of attention	2.95 ± 0.45	3.10 ± 0.53 ^¥^	0.30 (−0.53 to 1.14)			
	Differential learning	2.93 ± 0.45	3.44 ± 0.46 ^¥^	1.12 ^£^ (0.17 to 2.06)			
Peak hip external rotation moment (Nm/kg)	Control	1.47 ± 0.37	1.45 ± 0.37	0.05 (−0.88 to 0.78)	F = 66.135 *p* = 0.001 *	F = 1.648 *p* = 0.194	F = 42.768 *p* = 0.001 *
	Self-controlled feedback	1.40 ± 0.32	1.39 ± 0.28	0.03 (−0.91 to 0.84)			
	External focus of attention	1.36 ± 0.35	1.47 ± 29 ^¥^	0.34 (−0.49 to 1.18)			
	Differential learning	1.45 ± 0.45	1.97 ± 0.44 ^¥,a,b,c^	1.16 ^£^ (0.22 to 2.11)			
Peak knee abduction moment (Nm/kg)	Control	0.49 ± 0.12	0.49 ± 0.12	0.00 (−0.83 to 0.83)	F = 26.179 *p* = 0.001 *	F = 0.331 *p* = 0.803	F = 4.962 *p* = 0.005 *
	Self-controlled feedback	0.48 ± 0.11	0.42 ± 0.09 ^¥^	0.59 (−1.49 to 0.29)			
	External focus of attention	0.49 ± 0.07	0.43 ± 0.04 ^¥^	1.05 (−1.94 to −0.16)			
	Differential learning	0.53 ± 0.11	0.41 ± 0.10 ^¥^	1.14 (−2.08 to −0.19)			

Abbreviations: Results are presented as mean ± SD; *, statistically significant difference (*p* < 0.05); ^¥^, pretest to posttest significant difference; ^†^, effect size (95% confidence intervals); ^£^, large Cohen’s d effect size (0.8); results of Bonferroni post hoc test: ^a^ = significant difference between differential learning and control groups; ^b^ = significant difference between differential learning and self-controlled feedback groups; ^c^ = significant difference between differential learning and external focus of attention groups.

## Data Availability

The datasets generated during and/or analyzed during the current study are available from the corresponding author on reasonable request.

## References

[1] Sanders T.L., Maradit Kremers H., Bryan A.J., Larson D.R., Dahm D.L., Levy B.A., Stuart M.J., Krych A.J. (2016). Incidence of anterior cruciate ligament tears and reconstruction: A 21-year population-based study. Am. J. Sports Med..

[2] Krosshaug T., Nakamae A., Boden B.P., Engebretsen L., Smith G., Slauterbeck J.R., Hewett T.E., Bahr R. (2007). Mechanisms of anterior cruciate ligament injury in basketball: Video analysis of 39 cases. Am. J. Sports Med..

[3] Cochrane J.L., Lloyd D.G., Buttfield A., Seward H., McGivern J. (2007). Characteristics of anterior cruciate ligament injuries in Australian football. J. Sci. Med. Sport.

[4] Welling W., Benjaminse A., Gokeler A., Otten B. (2016). Enhanced retention of drop vertical jump landing technique: A randomized controlled trial. Hum. Mov. Sci..

[5] Gokeler A., Neuhaus D., Benjaminse A., Grooms D.R., Baumeister J. (2019). Principles of motor learning to support neuroplasticity after ACL injury: Implications for optimizing performance and reducing risk of second ACL Injury. Sport Med..

[6] Øiestad B.E., Holm I., Engebretsen L., Aune A.K., Gunderson R., Risberg M.A. (2013). The prevalence of patellofemoral osteoarthritis 12 years after anterior cruciate ligament reconstruction. Knee Surg. Sports Traumatol. Arthrosc..

[7] Sasaki S., Nagano Y., Ichikawa H. (2018). Loading differences in single-leg landing in the forehand-and backhand-side courts after an overhead stroke in badminton: A novel tri-axial accelerometer research. J. Sports Sci..

[8] Leppänen M., Pasanen K., Kujala U.M., Vasankari T., Kannus P., Äyrämö S., Krosshaug T., Bahr R., Avela J., Perttunen J. (2017). Stiff landings are associated with increased ACL injury risk in young female basketball and floorball players. Am. J. Sports Med..

[9] Boden B.P., Dean G.S., Feagin J.A., Garrett W.E. (2000). Mechanisms of anterior cruciate ligament injury. Orthopedics.

[10] Kiapour A.M., Demetropoulos C.K., Kiapour A., Quatman C.E., Wordeman S.C., Goel V.K., Hewett T.E. (2016). Strain response of the anterior cruciate ligament to uniplanar and multiplanar loads during simulated landings: Implications for injury mechanism. Am. J. Sports Med..

[11] Montgomery C., Blackburn J., Withers D., Tierney G., Moran C., Simms C. (2018). Mechanisms of ACL injury in professional rugby union: A systematic video analysis of 36 cases. Br. J. Sports Med..

[12] Stuelcken M.C., Mellifont D.B., Gorman A.D., Sayers M.G.L. (2016). Mechanisms of anterior cruciate ligament injuries in elite women’s netball: A systematic video analysis. J. Sports Sci..

[13] Richardson M.C., Wilkinson A., Chesterton P., Evans W. (2020). Effect of sand on landing knee valgus during single-leg land and drop jump tasks: Possible implications for ACL injury prevention and rehabilitation. J. Sport Rehabil..

[14] Simoneau G.G., Wilk K.E. (2012). The challenge of return to sports for patients post-ACL reconstruction. J. Orthop. Sports Phys. Ther..

[15] Arundale A.J.H., Bizzini M., Giordano A., Hewett T.E., Logerstedt D.S., Mandelbaum B., Scalzitti D.A., Silvers-Granelli H., Snyder-Mackler L., Altman R.D. (2018). Exercise-based knee and anterior cruciate ligament injury prevention: Clinical practice guidelines linked to the international classification of functioning, disability and health from the academy of orthopaedic physical therapy and the American Academy of sports physical therapy. J. Orthop. Sport Phys. Ther..

[16] Padua D.A., DiStefano L.J., Hewett T.E., Garrett W.E., Marshall S.W., Golden G.M., Shultz S.J., Sigward S.M. (2018). National Athletic Trainers’ Association position statement: Prevention of anterior cruciate ligament injury. J. Athl. Train..

[17] Wulf G. (2013). Attentional focus and motor learning: A review of 15 years. Int. Rev. Sport Exerc. Psychol..

[18] Schmidt R.A., Lee T.D., Winstein C., Wulf G., Zelaznik H.N. (2018). Motor Control and Learning: A Behavioral Emphasis.

[19] Adams J.A. (1971). A closed-loop theory of motor learning. J. Mot. Behav..

[20] Piccoli A., Rossettini G., Cecchetto S., Viceconti A., Ristori D., Turolla A., Maselli F., Testa M. (2018). Effect of attentional focus instructions on motor learning and performance of patients with central nervous system and musculoskeletal disorders: A systematic review. J. Funct. Morphol. Kinesiol..

[21] Rossettini G., Testa M., Vicentini M., Manganotti P. (2017). The effect of different attentional focus instructions during finger movement tasks in healthy subjects: An exploratory study. BioMed Res. Int..

[22] Olsen O.E., Myklebust G., Engebretsen L., Holme I., Bahr R. (2005). Exercises to prevent lower limb injuries in youth sports: Cluster randomised controlled trial. BMJ.

[23] Benjaminse A., Gokeler A., Dowling A.V., Faigenbaum A., Ford K.R., Hewett T.E., Onate J.A., Otten B., Myer G.D. (2015). Optimization of the anterior cruciate ligament injury prevention paradigm: Novel feedback techniques to enhance motor learning and reduce injury risk. J. Orthop. Sport Phys. Ther..

[24] Benjaminse A., Otten B., Gokeler A., Diercks R.L., Lemmink K.A. (2017). Motor learning strategies in basketball players and its implications for ACL injury prevention: A randomized controlled trial. Knee Surg. Sports Traumatol. Arthrosc..

[25] Ghanati H.A., Letafatkar A., Almonroeder T.G., Rabiei P. (2022). Examining the influence of attentional focus on the effects of a neuromuscular training program in male athletes. J. Strength Cond. Res..

[26] Sakurada T., Yoshida M., Nagai K. (2021). Individual optimal attentional strategy in motor learning tasks characterized by steady-state somatosensory and visual evoked potentials. Front. Hum. Neurosci..

[27] Oftadeh S., Bahram A., Yaali R., Ghadiri F., Schöllhorn W.I. (2021). External Focus or Differential Learning: Is There an Additive Effect on Learning a Futsal Goal Kick?. Int. J. Environ. Res. Public Health.

[28] Schöllhorn W.I., Beckmann H., Davids K. (2010). Exploiting system fluctuations. Differential training in physical prevention and rehabilitation programs for health and exercise. Medicina.

[29] Fuchs P.X., Fusco A., Bell J.W., von Duvillard S.P., Cortis C., Wagner H. (2020). Effect of differential training on female volleyball spike-jump technique and performance. Int. J. Sports Physiol. Perform..

[30] Schöllhorn W.I., Mayer-Kress G., Newell K.M., Michelbrink M. (2009). Time scales of adaptive behavior and motor learning in the presence of stochastic perturbations. Hum. Mov. Sci..

[31] Schöllhorn W.I. (2000). Applications of systems dynamic principles to technique and strength training. Acta Acad. Olymp. Est..

[32] Ziegler M.D., Zhong H., Roy R.R., Edgerton V.R. (2010). Why variability facilitates spinal learning. J. Neurosci..

[33] Newell K.M. (2003). Schema theory (1975): Retrospectives and prospectives. Res. Q. Exerc. Sport.

[34] Tumer E.C., Brainard M.S. (2007). Performance variability enables adaptive plasticity of ‘crystallized’ adult birdsong. Nature.

[35] Dhawale A.K., Smith M.A., Ölveczky B.P. (2017). The role of variability in motor learning. Annu. Rev. Neurosci..

[36] Newell K.M., McDonald P.V. (1994). Learning to coordinate redundant biomechanical degrees of freedom. Interlimb Coordination.

[37] Bartlett R., Wheat J., Robins M. (2007). Is movement variability important for sports biomechanists?. Sport Biomech..

[38] Orangi B.M., Yaali R., Bahram A., Aghdasi M.T., van der Kamp J., Vanrenterghem J., Jones P.A. (2021). Motor learning methods that induce high practice variability reduce kinematic and kinetic risk factors of non-contact ACL injury. Hum. Mov. Sci..

[39] Schöllhorn W.I. (1999). Individualität-ein Vernachlässigter Parameter. Leistungssport.

[40] Ranganathan R., Newell K.M. (2013). Changing up the routine: Intervention-induced variability in motor learning. Exerc. Sport Sci. Rev..

[41] Schöllhorn W.I., Rizzi N., Slapšinskaitė-Dackevičienė A., Leite N. (2022). Always Pay Attention to Which Model of Motor Learning You Are Using. Int. J. Environ. Res. Public Health.

[42] Prokopenko M., Polani D., Ay N. (2014). On the cross-disciplinary nature of guided self-organisation. Guided Self-Organization: Inception.

[43] Swinnen S.P., Zelaznik H.N. (1996). Information Feedback for motor skill learning: A review. Advances in Motor Learning and Control.

[44] Silverman S. (1994). Communication and motor skill learning: What we learn from research in the gymnasium. Quest.

[45] Schöllhorn W.I., Hegen P., Davids K. (2012). The nonlinear nature of learning-A differential learning approach. Open Sports Sci. J..

[46] Savelsbergh G.J.P., Kamper W.J., Rabius J., De Koning J.J., Schöllhorn W.I. (2010). A new method to learn to start in speed skating: A differencial learning approach. Int. J. Sport Psychol..

[47] Sheikhi B., Letafatkar A., Thomas A.C., Ford K.R. (2021). Altered trunk and lower extremity movement coordination after neuromuscular training with and without external focus instruction: A randomized controlled trial. BMC Sports Sci. Med. Rehabil..

[48] Stensrud S., Myklebust G., Kristianslund E., Bahr R., Krosshaug T. (2011). Correlation between two-dimensional video analysis and subjective assessment in evaluating knee control among elite female team handball players. Br. J. Sports Med..

[49] Tamura A., Akasaka K., Otsudo T., Sawada Y., Okubo Y., Shiozawa J., Toda J., Zamada K. (2016). Fatigue alters landing shock attenuation during a single-leg vertical drop jump. Orthop. J. Sport Med..

[50] Cohen J. (1992). A power primer. Psychol. Bull..

[51] Gokeler A., Benjaminse A., Seil R., Kerkhoffs G., Verhagen E. (2018). Using principles of motor learning to enhance ACL injury prevention programs. Sport Orthop. Traumatol..

[52] Widenhoefer T.L., Miller T.M., Weigand M.S., Watkins E.A., Almonroeder T.G. (2019). Training rugby athletes with an external attentional focus promotes more automatic adaptions in landing forces. Sport Biomech..

[53] Diekfuss J.A., Rhea C.K., Schmitz R.J., Grooms D.R., Wilkins R.W., Slutsky A.B., Raisbeck L.D. (2019). The influence of attentional focus on balance control over seven days of training. J. Mot. Behav..

[54] Benjaminse A., Welling W., Otten B., Gokeler A. (2018). Transfer of improved movement technique after receiving verbal external focus and video instruction. Knee Surg. Sports Traumatol. Arthrosc..

[55] Boden B.P., Torg J.S., Knowles S.B., Hewett T.E. (2009). Video analysis of anterior cruciate ligament injury: Abnormalities in hip and ankle kinematics. Am. J. Sports Med..

[56] Harty C.M., DuPont C.E., Chmielewski T.L., Mizner R.L. (2011). Intertask comparison of frontal plane knee position and moment in female athletes during three distinct movement tasks. Scand. J. Med. Sci. Sports.

[57] Fong C.-M., Blackburn J.T., Norcross M.F., McGrath M., Padua D.A. (2011). Ankle-dorsiflexion range of motion and landing biomechanics. J. Athl. Train..

[58] Shultz S.J., Schmitz R.J., Benjaminse A., Collins M., Ford K., Kulas A.S. (2015). ACL Research Retreat VII: An update on anterior cruciate ligament injury risk factor identification, screening, and prevention: March 19–21, 2015; Greensboro, NC. J. Athl. Train..

[59] Blackburn J.T., Padua D.A. (2009). Sagittal-plane trunk position, landing forces, and quadriceps electromyographic activity. J. Athl. Train..

[60] Khayambashi K., Ghoddosi N., Straub R.K., Powers C.M. (2016). Hip muscle strength predicts noncontact anterior cruciate ligament injury in male and female athletes: A prospective study. Am. J. Sports Med..

[61] Bates N.A., Nesbitt R.J., Shearn J.T., Myer G.D., Hewett T.E. (2017). Knee abduction affects greater magnitude of change in ACL and MCL strains than matched internal tibial rotation in vitro. Clin. Orthop. Relat. Res..

[62] Kiapour A.M., Kiapour A., Goel V.K., Quatman C.E., Wordeman S.C., Hewett T.E., Demetropoulos C.K. (2015). Uni-directional coupling between tibiofemoral frontal and axial plane rotation supports valgus collapse mechanism of ACL injury. J. Biomech..

[63] McNair P.J., Marshall R.N., Matheson J.A. (1990). Important features associated with acute anterior cruciate ligament injury. N. Z. Med. J..

[64] Chomiak J., Junge A., Peterson L., Dvorak J. (2000). Severe injuries in football players. Am. J. Sports Med..

